# Lectures on Anatomy, Interspersed with Practical Remarks

**Published:** 1831-01-01

**Authors:** 


### XIII.

LECTURES ON ANATOMY; INTERSPERSED WITH PRACTICAL REMARKS. By *Bransby B. Cooper*, F. R. S., Surgeon of Guy's Hospital, and Lecturer on Anatomy, &c. &c. Vol. II., large 8vo. pp. 308, two Plates. Highley, London, 1830.

WORKS on anatomy do not usually form the materials for reviews. An analysis would be preposterous, a critique ridiculous; for all that is required at the hands of the author is strict fidelity and minuteness, and a tolerably clear arrangement. Anatomical works may be divided into three classes. First, we may have a system of anatomy, and nothing else; no physiology, no surgery mixed up with the descriptive details. Secondly, we have the anatomical details interspersed with physiology, and practical precepts. And thirdly, we have the class of manuals, or dissectors, in which the textures of the body are not described apart and in series: but portions of the body, as the perineum for instance, are considered in the lump. Each has had its advocates. For our own parts, we approve, like Candide, of all. In dissecting a body the student must have a manual. In reading at home and at his leisure, of what he has dissected, he should possess a more systematic work. But here comes the question. Shall that systematic work be, like Cloquet's and Boyer's, confined to anatomical detail; or shall it, like most of the works in this country, and, among others, like Mr. Cooper's, be combined with physiology and surgery. We do not pretend to decide which plan is the best when both have their advantages. Perhaps such a work as Mr. Cooper's would be more acceptable to the majority of young men than Cloquet's or Boyer's; it is not so dry.

On a former occasion, we saw reason to speak in favourable terms of the first volume of the present work. We anticipated much from the zeal of the author, nor have we been mistaken. We have said, and we repeat it, that Bransby Cooper's Anatomy would become a very popular work. As a specimen of its mode of execution we subjoin a description of the cutis, taken, really, at random from the book. There may be many portions of a still better character, but when we cannot give an extended account of any book, the fairest mode of putting the public in possession of its merits or demerits, is thus to offer them a sample at hazard.

" The RETE MUCOSUM is situated immediately on the surface of the cutis, between it and the cuticle. The difficulty which attends the dissection and demonstration of this membrane, has given rise to various conjectures respecting its nature and particular structure. Hence the great variety of opinions between those physiologists who describe the rete mucosum as being composed of three distinct layers; while there are others of equal eminence, who deny its existence altogether. According to the result of my own investigation, the following structure appears to be demonstrable.—To shew the rete mucosum, a piece of healthy black skin, just removed from the body after an operation, should be immersed in boiling hot water, and then thrown into alcohol. The cuticle may now be carefully peeled off, leaving the rete mucosum on the surface of the cutis; and when this has been done, its structure is visible (*Vide Plate* II. *Fig.* 8.), and appears to be composed of a net-work of veins, assuming a peculiar inosculation, differing from that of arteries. It is characterized by frequent, short, anastomosing branches, uniting nearly at right angles, or rather forming figures in which right angles predominate; while, on the contrary, arterial inosculations form figures, in which acute angles predominate; and these are seen, in the minute injections of the arterial distribution, on the surface of the cutis. (*Vide Plate* II. *Fig.* 9.) The venous structure is situated immediately underneath the cuticle, and exterior to the minute arterial distribution on the surface of the cutis; and appears to be the true seat of colour of the skin. There are two preparations in which this structure is very successfully demonstrated; the one may be seen in the Museum of the College of Surgeons, and the other at Guy's Hospital; the representation, Plate II. Fig. 8, is taken from the latter, magnified in the proportion of one inch to one-eighth of an inch.

This structure appears to be much thicker in the negro than in the European, and the vessels themselves much larger. In the preparation above alluded to, in the College of Surgeons, it is so thick at one portion, as very much to resemble the rete mucosum of the cetacei.

There is considerable difficulty in separating the cuticle from the rete mucosum, in consequence of its firm adhesion; and in those preparations which are even most successful, portions of it will be seen adhering to the raised cuticle. The process of putrefaction seems to go on more readily in this structure than either in the cuticle or cutis, which occasions the ready separation of the cuticle, and probably explains the reason why, in this separation, the rete mucosum is not discernable, but merely a mucous deposit adhering both to the surface of the cutis and cuticle. These surfaces have, therefore, been described as separate layers; while the colouring matter, which is well known to exist, has been assigned to the space between them; thus giving rise to the supposition, that rete mucosum is formed of three separate layers of membrane.

As the seat of colour, the rete mucosum has an undeniable existence; and it is clear, that the cuticle possesses a semi-transparency, through which this colour is more or less discernable. The black colour of Negroes depends on the black colour of the rete mucosum, seen through the cuticle; in Europeans, also, various shades of colour are found, generally according with the colour of the hair, and eyes; these various shades may be observed in the different gradations between a fair and brunette's complexion; the former, generally with light blue; the latter, with dark eyes, and jet black hair. It is generally said, that the rete mucosum is wanting in the Albino, and in white animals of other species. It does not, however, accord with the usual operations of nature, to have any structure entirely wanting: it may exist in a colourless state, and in a very small quantity; still smaller than in the European, where it is evidently less than in the Negro.

Five principal shades have been enumerated as characteristic of the several races of mankind. In the Caucasian, white; in the Mongolian, yellow; in the Æthiopian, jet black; in the American, copper colour; in the Malayan, tawny, or resembling dark mahogany." 285.

It may readily be imagined that a work made up of such materials must be one of no mean value to the student, aye, and to the professional man in practice. When the whole of the volumes, four in number, are completed, we have no doubt whatever that their sale will amply prove the truth of our opinions.

